# The role of WNT and IL-1 signaling in osteoarthritis: therapeutic implications for platelet-rich plasma therapy

**DOI:** 10.3389/fragi.2023.1201019

**Published:** 2023-06-08

**Authors:** Antonio Tonutti, Valentina Granata, Veronica Marrella, Cristina Sobacchi, Rita Ragusa, Cristiano Sconza, Nicola Rani, Berardo Di Matteo, Angela Ceribelli

**Affiliations:** ^1^ Department of Biomedical Sciences, Humanitas University, Pieve Emanuele, Italy; ^2^ Rheumatology and Clinical Immunology, Istituti di Ricovero e Cura a Carattere Scientifico (IRCCS) Humanitas Research Hospital, Rozzano, Italy; ^3^ Human Genome and Biomedical Technologies Unit, Istituti di Ricovero e Cura a Carattere Scientifico (IRCCS) Humanitas Research Hospital, Rozzano, Italy; ^4^ Milan Unit, National Research Council—Institute for Genetic and Biomedical Research (CNR-IRGB), Milan, Italy; ^5^ Department of Rehabilitation and Functional Recovery, Istituti di Ricovero e Cura a Carattere Scientifico (IRCCS) Humanitas Research Hospital, Rozzano, Milan, Italy; ^6^ Conservative Orthopaedic Surgery and Innovative Techniques, Rizzoli Orthopaedic Institute, Bologna, Italy; ^7^ Division of Orthopedics, Istituti di Ricovero e Cura a Carattere Scientifico (IRCCS) Humanitas Research Hospital, Rozzano, Italy

**Keywords:** osteoarthritis, inflammatory pathways, platelet-rich-plasma, autologous therapy, immunology

## Abstract

Different from inflammatory arthritis, where biologicals and targeted synthetic molecules have revolutionized the disease course, no drug has demonstrated a disease modifying activity in osteoarthritis, which remains one of the most common causes of disability and chronic pain worldwide. The pharmacological therapy of osteoarthritis is mainly directed towards symptom and pain relief, and joint replacement is still the only curative strategy. Elucidating the disease pathophysiology is essential to understand which mechanisms can be targeted by innovative therapies. It has extensively been demonstrated that aberrant WNT and IL-1 signaling pathways are responsible for cartilage degeneration, impaired chondrocyte metabolism and differentiation, increased extracellular matrix degradation, and altered subchondral bone homeostasis. Platelet-rich plasma is an autologous blood derivative containing a concentration of platelets that is much higher than the whole blood counterpart and has shown promising results in the treatment of early knee osteoarthritis. Among the proposed mechanisms, the modulation of WNT and IL-1 pathways is of paramount importance and is herein reviewed in light of the proposed regenerative approaches.

## Introduction

Osteoarthritis (OA) is the most common disease affecting the joints, the prevalence exceeding 10% of the global population and constantly increasing (Glyn-Jones et al., 2015). Albeit almost any joint can be involved, the knee is one of the most frequently affected and symptomatic sites, with typical radiographic alterations being detectable in up to 37% of people over 60 years and associated symptoms in 12% of the global population (Dillon et al., 2006). Pain and disability deriving from OA represent major concerns, leading to a considerable economic burden, mainly in terms of indirect costs such as loss of productivity and informal care provided by caregivers (Leardini et al., 2004). Traditional risk factors for OA development include female gender, older age, elevated body mass index (i.e., overweight and obesity), mechanical factors (e.g., congenital hip dysplasia), and previous articular damage (e.g., sport injuries) (Sharma, 2021). However, due to the heterogeneity of OA localizations, differences in risk factors, pathogenic theories, clinical manifestations, and therapeutic strategies are being recognized in a site-specific manner (Kloppenburg et al., 2017). As an example, family history is a prominent risk factor for hand and hip OA (Haugen et al., 2020), whereas it is of limited importance in case of knee OA.

The diagnosis of OA relies on the clinical features, mainly mechanical joint pain and typical signs at physical examination (such as Heberden’s and Bouchard’s nodules in hand OA, trapeziometacarpal joint deformities, etc.) and is largely supported by imaging findings, particularly conventional radiography of the involved joints which remains the standard instrumental evaluation (Haugen and Bøyesen, 2011). Joint space narrowing, subchondral sclerosis, and osteophyte formation are suggestive elements when evaluating radiographs obtained in patients with OA (Swagerty and Hellinger, 2001). First-line treatments mostly rely on pharmacological systemic and intra-articular therapies, with pain relief as the most important objective; thus, these treatments only lead to short-term benefits whereas joint replacement surgery is the only resolutive strategy (Sharma, 2021). However, joint replacement is not free from complications since prosthetic joints have a limited life span and seldom require surgical revision (Sabah et al., 2021). Also, the risk of peri-prosthetic infection (Sharma, 2021) is associated to morbidity and mortality along with challenges and difficulties in diagnosis and management (Peel et al., 2012; Tande and Patel, 2014).

Taking these considerations into account, the treatment of OA is far from achieving the results that have been observed in other rheumatological subsets such as inflammatory arthritis (i.e., rheumatoid arthritis, spondyloarthritis) (Smolen et al., 2022; Ramiro et al., 2023); elucidating the disease pathogenesis is of utmost importance in order to discover effective (and hopefully permanent) “disease-modifying” treatment strategies. Disease modifying antirheumatic drugs (DMARDs) are a heterogeneous class of pharmaceuticals that intercept different but fundamental aspects in the pathogenesis of a disease, thus interfering and blocking the disease mechanisms. Similarly, a definition for “disease modifying OA drugs” (DMOADs) has been recently proposed (Oo and Hunter, 2022).

OA has been traditionally considered a mechanical and degenerative disorder, rather than an immune-mediated or inflammatory phenomenon (Vincent, 2019a); as a proof of concept, OA is usually counterposed to inflammatory arthritis (i.e., rheumatoid arthritis, spondyloarthritis, and microcrystalline arthritis) in both research models and clinical practice (Tu et al., 2023). A role for inflammatory cytokines, such as IL-1β and TNF-α, however, has been hypothesized in OA since the 1980s (Pujol and Loyau, 1987), so that in the English language the historical term “osteoarthrosis” (with the Latin suffix -*osis* standing for “degenerative process” without inflammation) (Atkinson, 1984) has been replaced by “osteoarthritis” to highlight the inflammatory component (Vincent, 2019a) that is part of the disease pathogenesis. Moreover, genetic polymorphisms and epigenetic modifications involving genes coding for inflammatory factors have been advocated and might help explain the family distribution that is typical of certain subsets of OA, such as the hand and the hip (Motta et al., 2022).

Platelet-rich plasma (PRP) is an autologous blood derivative containing a concentration of platelets that is much higher than the whole blood counterpart. PRP is enriched in molecules that are normally contained in platelet granules, including different cytokines and growth factors such as platelet-derived growth factor (PDGF), vascular endothelial growth factor (VEGF), insulin-like growth factor I (IGF-I), and transforming growth factor β (TGF-β), along with anti-inflammatory molecules (Rodríguez-Merchán, 2022). Such molecules play a relevant role in maintaining and restoring chondrocyte, synovial, and subchondral bone homeostasis (Bennell et al., 2017). Despite the unsatisfactory results achieved in two large randomized-controlled trials investigating knee and ankle OA (Bennell et al., 2017; Paget et al., 2021), a recent meta-analysis has suggested the superiority of PRP intra-articular injections compared to standard-of-care hyaluronic acid in terms of short-term functional recovery, joint functional improvement, and long-term pain relief (Tang et al., 2020; Belk et al., 2021). Also, PRP injection in a mouse model of early knee OA has been associated with a decreased incidence of radiological and symptomatic OA (Khatab et al., 2018). PRP administration has shown to improve the quality of life in patients with severe knee OA in a large clinical trial (Akan et al., 2018), whereas no significant benefit was reported in patients with mild and moderate radiographic damage (Bennell et al., 2021), thus suggesting that a correct timing of administration is crucial. Dosing of PRP is also of utmost importance, and a platelet count exceeding 10 billion is necessary to achieve sustained clinical benefits, along with a reduction in the amount of inflammatory cytokines in the synovial fluid (Bansal et al., 2021).

**FIGURE 1:**
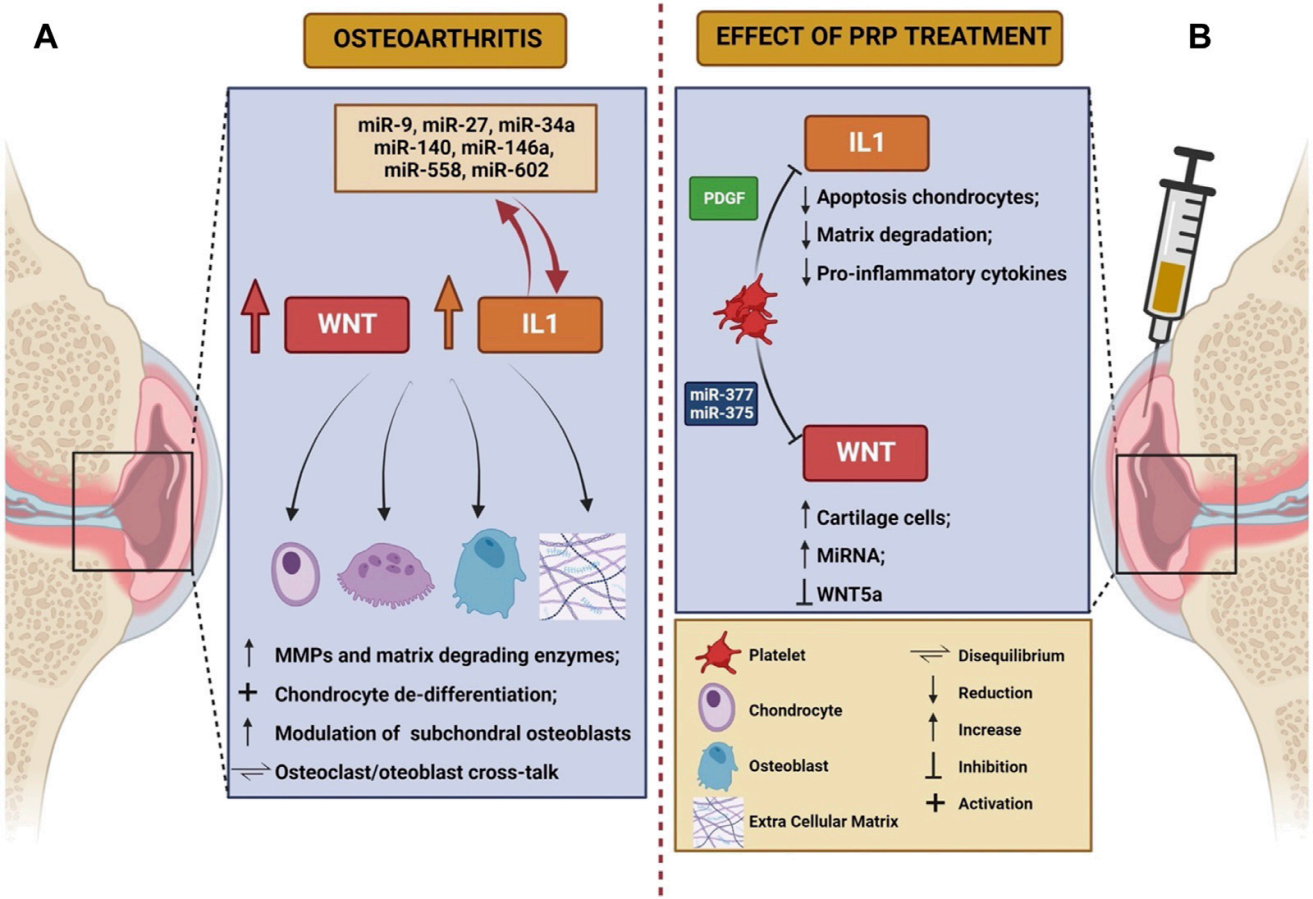
Schematic representation of the role of Wnt and IL-1 in the pathogenesis of osteoarthritis **(A)** and of the effects elicited on these mechanisms by PRP infusion **(B)**. The figure was created with BioRender.com (accessed on 24 March 2023).

Even though the exact mechanisms of action are largely unknown, anti-inflammatory and immune-modulating functions of PRP have been postulated (Bennell et al., 2017), along with disease modifying effects at both cartilage and synovial level (Boffa et al., 2021). Despite a large number of molecular mechanisms involved in OA have been elucidated (Moussa et al., 2017; Li et al., 2022; Yao et al., 2023), WNT signaling pathway and IL-1β-mediated signaling have gained importance in the disease pathogenesis during the last years. We will herein review the pathogenic significance of these mechanisms, with a focus on the possible roles of PRP in modulating such processes. The main characteristics of WNT and IL-1 pathway in the pathogenesis of knee OA, as well as the potential role of PRP in modulating their action, are summarized in Table 1 and schematically depicted in Figure 1.

**TABLE 1:** Executive summary of the characteristics of WNT and IL-1 signaling.

	WNT	IL-1β
Biologic function	Morphogenic growth factors	Inflammatory cytokine
Genetic defect consequences	Connective tissue abnormalities (skeleton, cartilage, teeth)	Autoinflammatory syndromes (*inflammasomopathies*)
Key transduction proteins	*Canonical pathway*	MAP kinase
	β-catenin	NF-κB
	*Noncanonical pathway*	Protein kinase C
	WNT-5a	Notch
Cellular effects	Chondrocyte dedifferentiation	Cartilage degradation
	ECM degradation	Cartilage fibrosis (⇧ type I collagen)
	Bone metabolism imbalance	Bone sclerosis
Effects of PRP	⇩ β-catenin	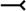 NF-κB
	⇩ TNF-α	⇩ inflammasome
	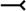 WNT-5a	⇧ IL-1Ra

Legenda: ⇧ stands for “increase”; ⇩ stands for “reduction”;
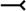
stands for “inhibition”.

## WNT signaling in OA

WNT is a conserved family of growth factors involved in the regulation of tissue development and differentiation (Clevers and Nusse, 2012). In particular, the morphogenic ability to shape tissues is one of the most important features distinguishing WNT from other families of growth factors (Clevers and Nusse, 2012). WNT signaling is involved in bone and joint formation since embryogenesis, contributing to the homeostasis of connective tissues during adult life (Hartmann and Tabin, 2001; Guo et al., 2004), including cartilage. Genetic defects in the WNT signal transduction pathway are indeed responsible for complex syndromes characterized by bone abnormalities and frailty, with defects in teeth and retinal development (Nusse and Clevers, 2017). More recently, polymorphisms in WNT genes have been linked to the predisposition towards OA development (Zhou et al., 2017), after observing that this pathway is overexpressed in the cartilage and synovium in both animal models and humans (De Santis et al., 2018).

Two major pathways of signal transduction have been described for proteins of the WNT family: the canonical (or β-catenin dependent) pathway, and the non-canonical pathway which is largely sustained by the WNT-5a isoform (Wang et al., 2019a). It has been described in animal models that aberrant canonical WNT signaling plays a critical role in OA pathogenesis: increased levels of β-catenin have been observed in the cartilage of mechanical stress-induced OA in rats (Liu et al., 2016), as well as in chondrocytes from mice stimulated with IL-1β (Bougault et al., 2014). It seems indeed that hyper-activation of canonical WNT signaling leads to chondrocyte overexpression of metalloproteinases (MMPs) and other extracellular matrix degrading enzymes (such as ADAMTS 4 and 5), with subsequent destruction of the cartilage (Blom et al., 2009). As for the non-canonical pathway, WNT-5a overexpression seems to promote changes leading to OA, such as cartilage degradation, synovial activation, and osteoclast/osteoblast activity imbalance in subchondral bone (Wang et al., 2019a). From a cellular point of view, hyper-activation of WNT in chondrocytes leads to their de-differentiation to mesenchymal cells with extracellular matrix degrading abilities (Yates et al., 2005). WNT silencing is essential instead during the development of osteo-chondroprogenitors to polarize their differentiation into matrix-producing chondrocytes (Yates et al., 2005), and WNT inhibition is capable to stimulate both chondrocyte proliferation and the synthesis of cartilage-specific collagen isoforms (i.e., type II collagen) (Kovács et al., 2019). Moreover, WNT antagonism modulates the activity of subchondral osteoblasts, thus reducing the formation of osteophytes: in this sense, despite not reaching satisfying results in preliminary studies (Yazici et al., 2020), lorecivivint (an inhibitor of the canonical WNT signaling pathway) is currently under investigation for the treatment of OA (Deshmukh et al., 2019; De Palma and Nalesso, 2021). Modulation of WNT signaling is thus a promising therapeutic target in patients suffering from OA, since both the β-catenin dependent and the non-canonical pathways drive the fundamental pathogenic mechanisms.

The pathogenic picture is however more complex, since it has been demonstrated that a balanced and appropriate degree of WNT signaling is required for cartilage homeostasis: both the complete inhibition and the hyper-expression of the canonical pathway have been associated to deleterious effects on chondral metabolism, with an increase in the risk of OA development in both animal and human models (Loughlin et al., 2004; Lories et al., 2007; Zhu et al., 2008; Zhu et al., 2009). Factors contributing to the regulation of such delicate equilibrium are largely unknown, but it has been postulated that the different WNT isoforms may exert distinct and even contrasting activities: as an example, despite WNT hyper-expression representing a well-established pathogenic factor in OA development, upregulation of WNT-16 is essential in preserving cartilage homeostasis following joint injury (Nalesso et al., 2017). More detailed information is required to elucidate the role of WNT in contributing to cartilage preservation *versus* degeneration, so that targeted inhibition of deleterious molecules and stimulation of protective isoforms could represent valid therapeutic strategies in the future.

## IL-1 signaling in OA

Patients with OA-related pain have increased serum levels of C-reactive protein compared to healthy controls, thus supporting the hypothesis that some degree of subclinical inflammation is a key contributor in the development and clinical manifestations of OA (Lane and Felson, 2020). IL-1 is a family of inflammatory cytokines, with soluble IL-1β being one of the most characterized elements (Migliorini et al., 2020), produced from the cleavage of inactive precursors through the action of inflammasomes, that are enzymatic complexes found in activated immune cells during the inflammatory response (Migliorini et al., 2020). Genetic defects leading to the constitutive activation of the inflammasome are responsible for a subset of autoinflammatory syndromes associated to excessive and dysregulated production of IL-1β, such as familial Mediterranean fever and mevalonate kinase deficiency syndrome (Lin and Goldbach-Mansky, 2022). The IL-1 system has been also advocated as a central actor in the pathogenesis of OA, and a close relationship between genetic polymorphisms of IL-1 and OA development has been described (Cai et al., 2015). IL-1β is among the most potent inducers of cartilage degradation (Vincent, 2019b), is capable of reducing the synthesis of type II collagen and proteoglycans, and can stimulate the release of matrix-degrading enzymes (MMPs, ADAMTS 4 and 5) (Kapoor et al., 2011) from chondrocytes. The CANTOS study was a randomized control trial investigating the role of canakinumab (a monoclonal antibody directed towards IL-1β) in secondary cardiovascular prevention; among secondary outcomes, it was observed that patients receiving canakinumab were less prone to undergo joint replacement surgery for knee OA compared to controls (Lane and Felson, 2020). Despite information on these outcomes was often nonspecifically reported or inconsistently collected, such results were confirmed even in the long-term (Lane and Felson, 2020). Despite the fact that the trial was not empowered to study the role of IL-1 inhibitors in OA, and was interrupted because of the increased infection risk, such data support the hypothesis of a critical role of cytokines, especially IL-1β, and inflammation in the pathogenesis of OA.

IL-1β exerts pleiotropic actions on multiple cells that are responsible for cartilage homeostasis, including chondrocytes, osteoblasts, osteoclasts, synovial macrophages, and fibroblasts (Jenei-Lanzl et al., 2019). Levels of membrane IL-1 receptor 1 (IL-1RI) as well as cytoplasmic proteins involved in IL-1β signaling transduction are upregulated in osteoarthritic chondrocytes (Martel-Pelletier et al., 1992; Ahmad et al., 2007). The action of IL-1β skews chondrocyte metabolism towards catabolism, thus inducing apoptosis and extracellular matrix degradation. Moreover, chondrocytes exposed to IL-1β acquire a fibroblast-like de-differentiated phenotype that results in the increase of type I collagen synthesis (i.e., the “fibrotic” collagen) at the expense of type II collagen, and upregulation of matrix proteinases (i.e., MMPs and ADAMTS) (Liacini et al., 2002), thus impairing the mechanical properties of the cartilage. The aforementioned deleterious changes are obtained through the interplay between IL-1β and different signaling pathways, including MAP kinase, NF-kB, protein kinase C, Notch, and even WNT (Jenei-Lanzl et al., 2019).

The IL-1 system can also alter bone cell metabolism, despite conflicting evidence has been reported. Following bone fractures, osteoblasts normally produce IL-1β and this cytokine is essential to boost the process of bone repair (Lin et al., 2010). Subchondral OA osteoblasts display the same ability to synthetize large amounts of IL-1β (Massicotte et al., 2002), which has been hypothesized to guide osteoproliferation, leading to osteophyte formation and subchondral bone sclerosis (Jenei-Lanzl et al., 2019). Notwithstanding, low-dose chronic exposure to IL-1β has shown to inhibit the synthetic functions of osteoblasts *in vitro*, and to induce a catabolic phenotype, for example, enhancing their expression of RANKL (Jenei-Lanzl et al., 2019). Furthermore, IL-1β modulates osteoclast metabolism, by inhibiting their apoptosis, and stimulating osteoclastogenesis (Jimi et al., 1998).

MicroRNAs (miRNA) play an important role in modulating IL-1β-induced OA damage (Sondag and Haqqi, 2016). High levels of miR-140 have been described in healthy chondrocytes, and are in turn significantly lower after chondrocytes are exposed *in vitro* to IL-1β, as well as in case of chondrocytes derived from osteoarthritic joints (Miyaki et al., 2009). A peculiar miRNA signature has been recently described in chondrocytes from patients with knee OA, and includes different molecules, such as miR-9, miR-27, miR-34a, miR-140, miR-146a, miR-558, and miR-602. Thus, non-coding RNAs are thought to be involved in the regulation of IL-1β-induced cartilage matrix degradation (O’Neill et al., 2011), and their signaling could represent an interesting therapeutic target for future research.

## PRP effects on IL-1 and WNT signaling

PRP has demonstrated clinical efficacy as a therapeutic strategy in a subgroup of patients affected by OA, by reducing the burden of symptoms and increasing the time-to-joint replacement especially in knee disease (Filardo et al., 2021). By containing a large amount of platelet-derived growth factors and cytokines, PRP is supposed to act by modifying the intra-articular cellular and molecular milieu (Figure 1B) but the pathogenetic mechanisms remain only partially understood, likely resulting in immune cell recruitment and induction of a regenerative response in both chondrocytes and synovial fibroblasts (Szwedowski et al., 2021). In particular, since PRP is composed of both pro-inflammatory and anti-inflammatory cytokines, it is thought that the interplay between these opposite forces can shift the balance of the osteoarthritic joint metabolism towards a favorable setting (Riewruja et al., 2022). However, detailed cytokine and growth factor profiling of PRP composition is needed to overcome the heterogeneity (Filardo et al., 2021) of previous evidence. It has been demonstrated that OA pathogenesis is characterized by a disproportion between classically activated pro-inflammatory macrophages (M1) and alternatively activated pro-healing macrophages (M2), in favor of the former population. PRP can restore the M1/M2 balance by re-polarizing M1 macrophages towards an M2 phenotype, by recruiting quiescent macrophages and blood monocytes in the OA joint and by inducing their polarization into an M2 type (Uchiyama et al., 2021).

PRP also reduces serum and joint fluid levels of pro-inflammatory cytokines such as IL-1β, IL-6, and VEGF (Sun et al., 2022), and can inhibit apoptosis in chondrocytes exposed to IL-1β, as well as extracellular matrix degradation (Yang et al., 2016). The effects of PRP on IL-1 system modulation are partially mediated by PDGF released from platelet granules (Montaseri et al., 2011), and a direct inhibitory effect of PRP on IL-1 transduction pathway has been postulated, with particular attention on the role of transcription factor NF-kB (Qi et al., 2021). Of note, similar molecular and immune results were observed with PRP in the conservative treatment of intervertebral disc degeneration where, along with the skewing of chondral macrophages towards an M2 phenotype, PRP showed to promote the degradation of NLRP3 inflammasome, with a subsequent reduction in levels of caspase-1 and IL-1β (Qian et al., 2022). PRP is also able to mitigate IL-1β inflammatory action by inducing an increase in intrarticular levels of IL-1 receptor antagonist (IL-1Ra), that is its natural decoy receptor and inhibitor (Barreto and Braun, 2016; Ziegler et al., 2019). In preclinical studies, IL-1Ra has indeed demonstrated to attenuate IL-1β-induced extracellular matrix degradation, also by restoring adequate autophagy processes (Wang et al., 2019b); meanwhile, plasma IL-1Ra levels have been negatively correlated with the risk of damage progression in a cohort of patients with early knee OA (Ma et al., 2020). It remains to be established whether baseline serum and synovial fluid levels of IL-1β, IL-1Ra, IL-6, and other inflammatory cytokines and biomarkers (including, e.g., C-reactive protein) could predict the therapeutic response to PRP injections.

The modulation of WNT/β-catenin signaling pathway is another proposed mechanism through which PRP is thought to exert its functions, thus suppressing apoptosis and inhibiting chondrocyte de-differentiation (Wu et al., 2018). In particular, PRP-derived exosomes are capable to inhibit the expression of WNT-5a, thus preventing chondrocyte death, and in turn inducing activation, proliferation and migration of cartilage cells (Liu et al., 2019). Increased levels of β-catenin, WNT-5a, and TNF-α have been described in IL-1β-treated chondrocytes; these phenomena are reversed after exposure to PRP-derived exosomes (Liu et al., 2019).

It was recently observed *in vitro* that PRP increases the expression of selected miRNAs, including miR-140, in mesenchymal stem cells (Konar et al., 2023). Also, miRNAs contained in PRP preparations can suppress inflammation and promote chondrocyte progenitors differentiation; it was indeed demonstrated that miR-337 and miR-375 contribute to OA alleviation through the aforementioned mechanisms (Sun et al., 2022). Notably, the same miRNAs were found to exert regulatory functions towards WNT signaling system in both cancer (Cui et al., 2018) and rheumatoid arthritis (Gui et al., 2015), thus inhibiting malignancy progression in the former case, and modulating the deleterious effects of activated synovial fibroblasts in the latter. We could thus postulate that PRP can contribute to restore joint homeostasis through a miRNA-dependent epigenetic regulation, which could constitute a novel therapeutic target for early forms of OA.

## From molecular immunology to clinical practice: limitations and unmet needs of PRP

Despite promising results, available clinical evidence is affected by much heterogeneity (Kon et al., 2020). First, high-quality RCT should report the preparation techniques and cellular composition of PRP (Kon et al., 2020). Second, due to the high variability in cytokine concentrations, data are poorly generalizable in clinical contexts (Ha et al., 2019; Kon et al., 2020). Third, a detailed profiling of selected patients is warranted, based on relevant comorbidities, age and sex, OA history (e.g., primary vs. post-traumatic, post-inflammatory, etc.) (Kon et al., 2020). Fourth, the correct timing of administration OF PRP needs yet to be established (Kon et al., 2020).

A precision medicine model is thus warranted, and molecular immunology represents the ideal tool to elucidate the role of PRP (and its detailed composition) in restoring the metabolic balance of OA joints, as well as to translate the acknowledged preclinical evidence into clinically significant results.

## Conclusion

Different from the progresses in the management of inflammatory arthritis, OA is considered an orphan disease, representing the main contributor to limitations in daily activities. By impairing walking in up to 20% of the affected subjects, OA has also been associated with an excess of overall mortality (Palazzo et al., 2016). Innovative treatments are required, but it is of outmost importance that the mechanisms underlying the disease pathogenesis are elucidated. The effects of PRP on the osteoarthritic joints sustain the hypothesis that the interplay between inflammatory and metabolic alterations drives the progression of OA and serves as prerequisite to further investigate biological and targeted therapies, however requiring more robust preclinical and clinical assumptions.

## References

[B1] Ahmad R. Sylvester J. Zafarullah M. (2007). MyD88, IRAK1 and TRAF6 knockdown in human chondrocytes inhibits interleukin-1-induced matrix metalloproteinase-13 gene expression and promoter activity by impairing MAP kinase activation. Cell. Signal. 19 (12), 2549–2557. 10.1016/j.cellsig.2007.08.013 17905570

[B2] Akan Ö. Sarıkaya N. Ö. Koçyiğit H. (2018). Efficacy of platelet-rich plasma administration in patients with severe knee osteoarthritis: Can platelet-rich plasma administration delay arthroplasty in this patient population? | cochrane library. Int. J. Clin. Exp. Med. 11, 9473–9483.

[B3] Atkinson M. H. (1984). Osteoarthrosis. Can. Fam. Physician. 30, 1503–1507. 21278961 PMC2153568

[B4] Bansal H. Leon J. Pont J. L. Wilson D. A. Bansal A. Agarwal D. (2021). Platelet-rich plasma (PRP) in osteoarthritis (OA) knee: Correct dose critical for long term clinical efficacy. Sci. Rep. 11 (1), 3971. 10.1038/s41598-021-83025-2 33597586 PMC7889864

[B5] Barreto A. Braun T. R. (2016). A method to induce Interleukin-1 Receptor Antagonist Protein from autologous whole blood. Cytokine 81, 137–141. 10.1016/j.cyto.2016.03.008 26994310

[B6] Belk J. W. Kraeutler M. J. Houck D. A. Goodrich J. A. Dragoo J. L. McCarty E. C. (2021). Platelet-rich plasma versus hyaluronic acid for knee osteoarthritis: A systematic review and meta-analysis of randomized controlled trials. Am. J. Sports Med. 49 (1), 249–260. 10.1177/0363546520909397 32302218

[B7] Bennell K. L. Hunter D. J. Paterson K. L. (2017). Platelet-rich plasma for the management of hip and knee osteoarthritis. Curr. Rheumatol. Rep. 19 (5), 24. 10.1007/s11926-017-0652-x 28386761

[B8] Bennell K. L. Paterson K. L. Metcalf B. R. Duong V. Eyles J. Kasza J. (2021). Effect of intra-articular platelet-rich plasma vs placebo injection on pain and medial tibial cartilage volume in patients with knee osteoarthritis: The RESTORE randomized clinical trial. JAMA 326 (20), 2021–2030. 10.1001/jama.2021.19415 34812863 PMC8611484

[B9] Blom A. B. Brockbank S. M. van Lent P. L. van Beuningen H. M. Geurts J. Takahashi N. (2009). Involvement of the Wnt signaling pathway in experimental and human osteoarthritis: Prominent role of wnt-induced signaling protein 1. Arthritis Rheum. 60 (2), 501–512. 10.1002/art.24247 19180479

[B10] Boffa A. Salerno M. Merli G. De Girolamo L. Laver L. Magalon J. (2021). Platelet-rich plasma injections induce disease-modifying effects in the treatment of osteoarthritis in animal models. Knee Surg. Sports Traumatol. Arthrosc. 29 (12), 4100–4121. 10.1007/s00167-021-06659-9 34341845

[B11] Bougault C. Priam S. Houard X. Pigenet A. Sudre L. Lories R. J. (2014). Protective role of frizzled-related protein B on matrix metalloproteinase induction in mouse chondrocytes. Arthritis Res. Ther. 16 (4), R137. 10.1186/ar4599 24984954 PMC4226985

[B12] Cai H. Sun H. J. Wang Y. H. Zhang Z. (2015). Relationships of common polymorphisms in IL-6, IL-1A, and IL-1B genes with susceptibility to osteoarthritis: A meta-analysis. Clin. Rheumatol. 34 (8), 1443–1453. 10.1007/s10067-014-2708-x 24952309

[B13] Clevers H. Nusse R. (2012). Wnt/β-catenin signaling and disease. Cell. 149 (6), 1192–1205. 10.1016/j.cell.2012.05.012 22682243

[B14] Cui H. Song R. Wu J. Wang W. Chen X. Yin J. (2018). MicroRNA-337 regulates the PI3K/AKT and Wnt/β-catenin signaling pathways to inhibit hepatocellular carcinoma progression by targeting high-mobility group AT-hook 2. Am. J. Cancer Res. 8 (3), 405–421. 29636997 PMC5883092

[B15] De Palma A. Nalesso G. (2021). WNT signalling in osteoarthritis and its pharmacological targeting. Handb. Exp. Pharmacol. 269, 337–356. 10.1007/164_2021_525 34510305

[B16] De Santis M. Di Matteo B. Chisari E. Cincinelli G. Angele P. Lattermann C. (2018). The role of Wnt pathway in the pathogenesis of OA and its potential therapeutic implications in the field of regenerative medicine. Biomed. Res. Int. 2018, 7402947. 10.1155/2018/7402947 30410938 PMC6205317

[B17] Deshmukh V. O’Green A. L. Bossard C. Seo T. Lamangan L. Ibanez M. (2019). Modulation of the Wnt pathway through inhibition of CLK2 and DYRK1A by lorecivivint as a novel, potentially disease-modifying approach for knee osteoarthritis treatment. Osteoarthr. Cartil. 27 (9), 1347–1360. 10.1016/j.joca.2019.05.006 31132406

[B18] Dillon C. F. Rasch E. K. Gu Q. Hirsch R. (2006). Prevalence of knee osteoarthritis in the United States: Arthritis data from the third national health and nutrition examination survey 1991-94. J. Rheumatol. 33 (11), 2271–2279. 17013996

[B19] Filardo G. Previtali D. Napoli F. Candrian C. Zaffagnini S. Grassi A. (2021). PRP injections for the treatment of knee osteoarthritis: A meta-analysis of randomized controlled trials. Cartilage 13 (1), 364S–375S. 10.1177/1947603520931170 32551947 PMC8808870

[B20] Glyn-Jones S. Palmer A. J. R. Agricola R. Price A. J. Vincent T. L. Weinans H. (2015). Osteoarthr. Lancet 386 (9991), 376–387. 10.1016/S0140-6736(14)60802-3 25748615

[B21] Gui M. C. jing S. W. Xiongyi Y. Yu H. Zhanglin X. Qinsong M. (2015). miR-375 regulates the canonical Wnt pathway through FZD8 silencing in arthritis synovial fibroblasts. Immunol. Lett. 164 (1), 1–10. 10.1016/j.imlet.2015.01.003 25619565

[B22] Guo X. Day T. F. Jiang X. Garrett-Beal L. Topol L. Yang Y. (2004). Wnt/beta-catenin signaling is sufficient and necessary for synovial joint formation. Genes. Dev. 18 (19), 2404–2417. 10.1101/gad.1230704 15371327 PMC522990

[B23] Ha C. W. Park Y. B. Jang J. W. Kim M. Kim J. A. Park Y. G. (2019). Variability of the composition of growth factors and cytokines in platelet-rich plasma from the knee with osteoarthritis. Arthroscopy 35 (10), 2878–2884. 10.1016/j.arthro.2019.04.010 31604507

[B24] Hartmann C. Tabin C. J. (2001). Wnt-14 plays a pivotal role in inducing synovial joint formation in the developing appendicular skeleton. Cell. 104 (3), 341–351. 10.1016/s0092-8674(01)00222-7 11239392

[B25] Haugen I. K. Bøyesen P. (2011). Imaging modalities in hand osteoarthritis-and perspectives of conventional radiography, magnetic resonance imaging, and ultrasonography. Arthritis Res. Ther. 13 (6), 248. 10.1186/ar3509 22189142 PMC3334630

[B26] Haugen I. K. Felson D. T. Abhishek A. Berenbaum F. Bierma-Zeinstra S. Borgen T. (2020). Development of classification criteria for hand osteoarthritis: Comparative analyses of persons with and without hand osteoarthritis. RMD Open 6 (2), e001265. 10.1136/rmdopen-2020-001265 32584781 PMC7425183

[B27] Jenei-Lanzl Z. Meurer A. Zaucke F. (2019). Interleukin-1β signaling in osteoarthritis - chondrocytes in focus. Cell. Signal 53, 212–223. 10.1016/j.cellsig.2018.10.005 30312659

[B28] Jimi E. Nakamura I. Ikebe T. Akiyama S. Takahashi N. Suda T. (1998). Activation of NF-kappaB is involved in the survival of osteoclasts promoted by interleukin-1. J. Biol. Chem. 273 (15), 8799–8805. 10.1074/jbc.273.15.8799 9535858

[B29] Kapoor M. Martel-Pelletier J. Lajeunesse D. Pelletier J. P. Fahmi H. (2011). Role of proinflammatory cytokines in the pathophysiology of osteoarthritis. Nat. Rev. Rheumatol. 7 (1), 33–42. 10.1038/nrrheum.2010.196 21119608

[B30] Khatab S. van Buul G. M. Kops N. Bastiaansen-Jenniskens Y. M. Bos P. K. Verhaar J. A. (2018). Intra-articular injections of platelet-rich plasma releasate reduce pain and synovial inflammation in a mouse model of osteoarthritis. Am. J. Sports Med. 46 (4), 977–986. 10.1177/0363546517750635 29373806

[B31] Kloppenburg M. van Beest S. Kroon F. P. B. (2017). Thumb base osteoarthritis: A hand osteoarthritis subset requiring a distinct approach. Best. Pract. Res. Clin. Rheumatol. 31 (5), 649–660. 10.1016/j.berh.2018.08.007 30509411

[B32] Kon E. Di Matteo B. Delgado D. Cole B. J. Dorotei A. Dragoo J. L. (2020). Platelet-rich plasma for the treatment of knee osteoarthritis: An expert opinion and proposal for a novel classification and coding system. Expert Opin. Biol. Ther. 20 (12), 1447–1460. 10.1080/14712598.2020.1798925 32692595

[B33] Konar E. Khatami S. R. Pezeshki S. P. Shafiei M. Hajjari M. R. (2023). The effect of PRP and hyperosmolarity simultaneous use on expression profile alteration of miRNAs associated with cartilage differentiation in human adipose tissue-derived mesenchymal stem cells. Gene 859, 147188. 10.1016/j.gene.2023.147188 36632912

[B34] Kovács B. Vajda E. Nagy E. E. (2019). Regulatory effects and interactions of the Wnt and OPG-RANKL-RANK signaling at the bone-cartilage interface in osteoarthritis. Int. J. Mol. Sci. 20 (18), 4653. 10.3390/ijms20184653 31546898 PMC6769977

[B35] Lane N. Felson D. (2020). A promising treatment for osteoarthritis? Ann. Intern Med. 173 (7), 580–581. 10.7326/M20-4938 32744863 PMC10804980

[B36] Leardini G. Salaffi F. Caporali R. Canesi B. Rovati L. Montanelli R. (2004). Direct and indirect costs of osteoarthritis of the knee. Clin. Exp. Rheumatol. 22 (6), 699–706. 15638043

[B37] Li M. Han H. Chen L. Li H. (2022). Platelet-rich plasma contributes to chondroprotection by repairing mitochondrial function via AMPK/NF-κB signaling in osteoarthritic chondrocytes. Tissue Cell. 77, 101830. 10.1016/j.tice.2022.101830 35644053

[B38] Liacini A. Sylvester J. Li W. Q. Zafarullah M. (2002). Inhibition of interleukin-1-stimulated MAP kinases, activating protein-1 (AP-1) and nuclear factor kappa B (NF-κB) transcription factors down-regulates matrix metalloproteinase gene expression in articular chondrocytes. Matrix Biol. 21 (3), 251–262. 10.1016/s0945-053x(02)00007-0 12009331

[B39] Lin B. Goldbach-Mansky R. (2022). Pathogenic insights from genetic causes of autoinflammatory inflammasomopathies and interferonopathies. J. Allergy Clin. Immunol. 149 (3), 819–832. 10.1016/j.jaci.2021.10.027 34893352 PMC8901451

[B40] Lin F. H. Chang J. B. McGuire M. H. Yee J. A. Brigman B. E. (2010). Biphasic effects of interleukin-1beta on osteoblast differentiation *in vitro* . J. Orthop. Res. 28 (7), 958–964. 10.1002/jor.21099 20108347

[B41] Liu S. S. Zhou P. Zhang Y. (2016). Abnormal expression of key genes and proteins in the canonical Wnt/β-catenin pathway of articular cartilage in a rat model of exercise-induced osteoarthritis. Mol. Med. Rep. 13 (3), 1999–2006. 10.3892/mmr.2016.4798 26794964 PMC4768959

[B42] Liu X. Wang L. Ma C. Wang G. Zhang Y. Sun S. (2019). Exosomes derived from platelet-rich plasma present a novel potential in alleviating knee osteoarthritis by promoting proliferation and inhibiting apoptosis of chondrocyte via Wnt/β-catenin signaling pathway. J. Orthop. Surg. Res. 14 (1), 470. 10.1186/s13018-019-1529-7 31888697 PMC6936129

[B43] Lories R. J. U. Peeters J. Bakker A. Tylzanowski P. Derese I. Schrooten J. (2007). Articular cartilage and biomechanical properties of the long bones in Frzb-knockout mice. Arthritis Rheum. 56 (12), 4095–4103. 10.1002/art.23137 18050203

[B44] Loughlin J. Dowling B. Chapman K. Marcelline L. Mustafa Z. Southam L. (2004). Functional variants within the secreted frizzled-related protein 3 gene are associated with hip osteoarthritis in females. Proc. Natl. Acad. Sci. U. S. A. 101 (26), 9757–9762. 10.1073/pnas.0403456101 15210948 PMC470747

[B45] Ma C. A. Rajandran S. N. Liu J. Wong S. B. S. Leung Y. Y. (2020). The association of plasma IL-1Ra and related cytokines with radiographic severity of early knee osteoarthritis. Osteoarthr. Cartil. Open 2 (2), 100046. 10.1016/j.ocarto.2020.100046 36474587 PMC9718200

[B46] Martel-Pelletier J. Mccollum R. Dibattista J. Faure M. P. Chin J. A. Fournier S. (1992). The interleukin-1 receptor in normal and osteoarthritic human articular chondrocytes. Identification as the type I receptor and analysis of binding kinetics and biologic function. Arthritis & Rheumatism. 35 (5), 530–540. 10.1002/art.1780350507 1533521

[B47] Massicotte F. Lajeunesse D. Benderdour M. Pelletier J. P. Hilal G. Duval N. (2002). Can altered production of interleukin-1beta, interleukin-6, transforming growth factor-beta and prostaglandin E(2) by isolated human subchondral osteoblasts identify two subgroups of osteoarthritic patients. Osteoarthr. Cartil. 10 (6), 491–500. 10.1053/joca.2002.0528 12056853

[B48] Migliorini P. Italiani P. Pratesi F. Puxeddu I. Boraschi D. (2020). The IL-1 family cytokines and receptors in autoimmune diseases. Autoimmun. Rev. 19 (9), 102617. 10.1016/j.autrev.2020.102617 32663626

[B49] Miyaki S. Nakasa T. Otsuki S. Grogan S. P. Higashiyama R. Inoue A. (2009). MicroRNA-140 is expressed in differentiated human articular chondrocytes and modulates interleukin-1 responses. Arthritis Rheum. 60 (9), 2723–2730. 10.1002/art.24745 19714579 PMC2806094

[B50] Montaseri A. Busch F. Mobasheri A. Buhrmann C. Aldinger C. Rad J. S. (2011). IGF-1 and PDGF-bb suppress IL-1β-induced cartilage degradation through down-regulation of NF-κB signaling: Involvement of src/PI-3K/AKT pathway. PLoS One 6 (12), e28663. 10.1371/journal.pone.0028663 22194879 PMC3237481

[B51] Motta F. Barone E. Sica A. Selmi C. (2022). Inflammaging and osteoarthritis. Clin. Rev. Allerg. Immunol. 64, 222–238. 10.1007/s12016-022-08941-1 35716253

[B52] Moussa M. Lajeunesse D. Hilal G. El Atat O. Haykal G. Serhal R. (2017). Platelet rich plasma (PRP) induces chondroprotection via increasing autophagy, anti-inflammatory markers, and decreasing apoptosis in human osteoarthritic cartilage. Exp. Cell. Res. 352 (1), 146–156. 10.1016/j.yexcr.2017.02.012 28202394

[B53] Nalesso G. Thomas B. L. Sherwood J. C. Yu J. Addimanda O. Eldridge S. E. (2017). WNT16 antagonises excessive canonical WNT activation and protects cartilage in osteoarthritis. Ann. Rheum. Dis. 76 (1), 218–226. 10.1136/annrheumdis-2015-208577 27147711 PMC5264226

[B54] Nusse R. Clevers H. (2017). Wnt/β-Catenin signaling, disease, and emerging therapeutic modalities. Cell. 169 (6), 985–999. 10.1016/j.cell.2017.05.016 28575679

[B55] O’Neill L. A. Sheedy F. J. McCoy C. E. (2011). MicroRNAs: The fine-tuners of toll-like receptor signalling. Nat. Rev. Immunol. 11 (3), 163–175. 10.1038/nri2957 21331081

[B56] Oo W. M. Hunter D. J. (2022). Repurposed and investigational disease-modifying drugs in osteoarthritis (DMOADs). Ther. Adv. Musculoskelet. Dis. 14, 1759720X221090297. 10.1177/1759720X221090297 PMC9128067 35619876

[B57] Paget L. D. A. Reurink G. de Vos R. J. Weir A. Moen M. H. Bierma-Zeinstra S. M. A. (2021). Effect of platelet-rich plasma injections vs placebo on ankle symptoms and function in patients with ankle osteoarthritis: A randomized clinical trial. JAMA 326 (16), 1595–1605. 10.1001/jama.2021.16602 34698782 PMC8548954

[B58] Palazzo C. Nguyen C. Lefevre-Colau M. M. Rannou F. Poiraudeau S. (2016). Risk factors and burden of osteoarthritis. Ann. Phys. Rehabil. Med. 59 (3), 134–138. 10.1016/j.rehab.2016.01.006 26904959

[B59] Peel T. N. Buising K. L. Choong P. F. M. (2012). Diagnosis and management of prosthetic joint infection. Curr. Opin. Infect. Dis. 25 (6), 670–676. 10.1097/QCO.0b013e32835915db 22964949

[B60] Pujol J. P. Loyau G. (1987). Interleukin-1 and osteoarthritis. Life Sci. 41 (10), 1187–1198. 10.1016/0024-3205(87)90196-2 3306235

[B61] Qi Y. Tang R. Shi Z. Feng G. Zhang W. (2021). Wnt5a/Platelet-rich plasma synergistically inhibits IL-1β-induced inflammatory activity through NF-κB signaling pathway and prevents cartilage damage and promotes meniscus regeneration. J. Tissue Eng. Regen. Med. 15 (7), 612–624. 10.1002/term.3198 33843153

[B62] Qian J. Wang X. Su G. Shu X. Huang Z. Jiang H. (2022). Platelet-rich plasma-derived exosomes attenuate intervertebral disc degeneration by promoting NLRP3 autophagic degradation in macrophages. Int. Immunopharmacol. 110, 108962. 10.1016/j.intimp.2022.108962 35753124

[B63] Ramiro S. Nikiphorou E. Sepriano A. Ortolan A. Webers C. Baraliakos X. (2023). ASAS-EULAR recommendations for the management of axial spondyloarthritis: 2022 update. Ann. Rheum. Dis. 82 (1), 19–34. 10.1136/ard-2022-223296 36270658

[B64] Riewruja K. Phakham S. Sompolpong P. Reantragoon R. Tanavalee A. Ngarmukos S. (2022). Cytokine profiling and intra-articular injection of autologous platelet-rich plasma in knee osteoarthritis. Int. J. Mol. Sci. 23 (2), 890. 10.3390/ijms23020890 35055075 PMC8779764

[B65] Rodríguez-Merchán E. C. (2022). Intra-articular platelet-rich plasma injections in knee osteoarthritis: A review of their current molecular mechanisms of action and their degree of efficacy. Int. J. Mol. Sci. 23 (3), 1301. 10.3390/ijms23031301 35163225 PMC8836227

[B66] Sabah S. A. Alvand A. Price A. J. (2021). Revision knee replacement for prosthetic joint infection: Epidemiology, clinical outcomes and health-economic considerations. Knee 28, 417–421. 10.1016/j.knee.2020.12.024 33500184

[B67] Sharma L. (2021). Osteoarthritis of the knee. N. Engl. J. Med. 384 (1), 51–59. 10.1056/nejmcp1903768 33406330

[B68] Smolen J. S. Landewé R. B. M. Bergstra S. A. Kerschbaumer A. Sepriano A. Aletaha D. (2022). EULAR recommendations for the management of rheumatoid arthritis with synthetic and biological disease-modifying antirheumatic drugs: 2022 update. Ann. Rheumatic Dis. 82, 3–18. 10.1136/ard-2022-223356 36357155

[B69] Sondag G. R. Haqqi T. M. (2016). The role of MicroRNAs and their targets in osteoarthritis. Curr. Rheumatol. Rep. 18 (8), 56. 10.1007/s11926-016-0604-x 27402113 PMC5294969

[B70] Sun X. Mi L. Du G. Sun C. He S. (2022). Platelet-rich plasma treatment alleviates osteoarthritis-related pain, inflammation, and apoptosis by upregulating the expression levels of microRNA-375 and microRNA-337. Immunopharmacol. Immunotoxicol. 44 (1), 87–98. 10.1080/08923973.2021.2007263 34845965

[B71] Swagerty D. L. Hellinger D. (2001). Radiographic assessment of osteoarthritis. Am. Fam. Physician 64 (2), 279–286. 11476273

[B72] Szwedowski D. Szczepanek J. Paczesny Ł. Zabrzyński J. Gagat M. Mobasheri A. (2021). The effect of platelet-rich plasma on the intra-articular microenvironment in knee osteoarthritis. Int. J. Mol. Sci. 22 (11), 5492. 10.3390/ijms22115492 34071037 PMC8197096

[B73] Tande A. J. Patel R. (2014). Prosthetic joint infection. Clin. Microbiol. Rev. 27 (2), 302–345. 10.1128/CMR.00111-13 24696437 PMC3993098

[B74] Tang J. Z. Nie M. J. Zhao J. Z. Zhang G. C. Zhang Q. Wang B. (2020). Platelet-rich plasma versus hyaluronic acid in the treatment of knee osteoarthritis: A meta-analysis. J. Orthop. Surg. Res. 15 (1), 403. 10.1186/s13018-020-01919-9 32912243 PMC7488405

[B75] Tu J. Chen W. Fang Y. Han D. Chen Y. Jiang H. (2023). PU.1 promotes development of rheumatoid arthritis via repressing FLT3 in macrophages and fibroblast-like synoviocytes. Ann. Rheum. Dis. 82 (2), 198–211. 10.1136/ard-2022-222708 36198439 PMC9887374

[B76] Uchiyama R. Toyoda E. Maehara M. Wasai S. Omura H. Watanabe M. (2021). Effect of platelet-rich plasma on M1/M2 macrophage polarization. Int. J. Mol. Sci. 22 (5), 2336. 10.3390/ijms22052336 33652994 PMC7956636

[B77] Vincent T. L. (2019). IL-1 in osteoarthritis: Time for a critical review of the literature. F1000Res 8, 934. 10.12688/f1000research.18831.1 PMC6589928 31249675

[B78] Vincent T. L. (2019). Mechanoflammation in osteoarthritis pathogenesis. Semin. Arthritis Rheum. 49 (3S), S36–S38. 10.1016/j.semarthrit.2019.09.018 31779850

[B79] Wang F. Liu J. Chen X. Zheng X. Qu N. Zhang B. (2019). IL-1β receptor antagonist (IL-1Ra) combined with autophagy inducer (TAT-Beclin1) is an effective alternative for attenuating extracellular matrix degradation in rat and human osteoarthritis chondrocytes. Arthritis Res. Ther. 21 (1), 171. 10.1186/s13075-019-1952-5 31291980 PMC6617669

[B80] Wang Y. Fan X. Xing L. Tian F. (2019). Wnt signaling: A promising target for osteoarthritis therapy. Cell. Commun. Signal 17 (1), 97. 10.1186/s12964-019-0411-x 31420042 PMC6697957

[B81] Wu J. Huang J. F. Qin X. X. Hu F. Chen Z. F. Zheng Y. (2018). Platelet-rich plasma inhibits Wnt/β-catenin signaling in rabbit cartilage cells activated by IL-1β. Int. Immunopharmacol. 55, 282–289. 10.1016/j.intimp.2017.12.031 29291543

[B82] Yang J. Lu Y. Guo A. (2016). Platelet-rich plasma protects rat chondrocytes from interleukin-1β-induced apoptosis. Mol. Med. Rep. 14 (5), 4075–4082. 10.3892/mmr.2016.5767 27665780 PMC5101884

[B83] Yao Q. Wu X. Tao C. Gong W. Chen M. Qu M. (2023). Osteoarthritis: Pathogenic signaling pathways and therapeutic targets. Signal Transduct. Target Ther. 8 (1), 56. 10.1038/s41392-023-01330-w 36737426 PMC9898571

[B84] Yates K. E. Shortkroff S. Reish R. G. (2005). Wnt influence on chondrocyte differentiation and cartilage function. DNA Cell. Biol. 24 (7), 446–457. 10.1089/dna.2005.24.446 16008513

[B85] Yazici Y. McAlindon T. E. Gibofsky A. Lane N. E. Clauw D. Jones M. (2020). Lorecivivint, a novel intraarticular CDC-like kinase 2 and dual-specificity tyrosine phosphorylation-regulated kinase 1A inhibitor and Wnt pathway modulator for the treatment of knee osteoarthritis: A phase II randomized trial. Arthritis Rheumatol. 72 (10), 1694–1706. 10.1002/art.41315 32432388 PMC7589351

[B86] Zhou Y. Wang T. Hamilton J. L. Chen D. (2017). Wnt/β-catenin signaling in osteoarthritis and in other forms of arthritis. Curr. Rheumatol. Rep. 19 (9), 53. 10.1007/s11926-017-0679-z 28752488 PMC5672801

[B87] Zhu M. Chen M. Zuscik M. Wu Q. Wang Y. J. Rosier R. N. (2008). Inhibition of beta-catenin signaling in articular chondrocytes results in articular cartilage destruction. Arthritis Rheum. 58 (7), 2053–2064. 10.1002/art.23614 18576323 PMC2667964

[B88] Zhu M. Tang D. Wu Q. Hao S. Chen M. Xie C. (2009). Activation of beta-catenin signaling in articular chondrocytes leads to osteoarthritis-like phenotype in adult beta-catenin conditional activation mice. J. Bone Min. Res. 24 (1), 12–21. 10.1359/jbmr.080901 PMC2640321 18767925

[B89] Ziegler C. G. Van Sloun R. Gonzalez S. Whitney K. E. DePhillipo N. N. Kennedy M. (2019). Characterization of growth factors, cytokines and chemokines in bone marrow concentrate and platelet rich plasma: A prospective analysis. Orthop. J. Sports Med. 7 (75), 2174–2187. 10.1177/0363546519832003 31034242

